# Efficacy and safety of the thumbtack needle for neck pain: a systematic review and meta-analysis

**DOI:** 10.3389/fpain.2025.1687334

**Published:** 2026-01-12

**Authors:** Jinyi He, Peng Lai, Xingyao Chen, Jiacheng Liu, Ziyu Wang, Chenyang Jia, Yu Liu, Shirui Cheng

**Affiliations:** 1 Acupuncture and Tuina School, Chengdu University of Traditional Chinese Medicine, Chengdu, China; 2 Acupuncture & Brain Research Center, Chengdu University of Traditional Chinese Medicine, Chengdu, China; 3 Key Laboratory of Acupuncture for Senile Disease (Chengdu University of TCM), Ministry of Education, Chengdu, China; 4 Hospital of Chengdu University of Traditional Chinese Medicine, School of Clinical Medicine, Chengdu University of Traditional Chinese Medicine, Chengdu, China

**Keywords:** thumbtack needle, neck pain, meta-analysis, systematic review, quality evaluation

## Abstract

**Background:**

Neck pain (NP) is a common musculoskeletal disorder that significantly affects the physical function and quality of life of patients. Thumbtack needle therapy is widely used to manage NP. However, previous studies have reported inconsistent clinical outcomes. This study aims to systematically evaluate the efficacy and safety of thumbtack needle therapy for NP.

**Methods:**

A systematic search was conducted in the Cochrane Library, Web of Science, Embase, PubMed, China National Knowledge Infrastructure (CNKI), China Science and Technology Journal (VIP), and Wanfang databases from their inception to 24 September 2023 for randomized controlled trials (RCTs) on thumbtack needle therapy for NP. Outcome measures included the visual analog scale (VAS) scores, neck disability index (NDI) scores, total effective rate, and adverse events. A meta-analysis was performed using Review Manager 5.3. The quality of evidence was assessed using the Grading of Recommendations Assessment, Development, and Evaluation (GRADE) system.

**Results:**

Seven RCTs involving 425 patients were included. Compared with the control group, thumbtack needle therapy significantly reduced VAS scores (MD = −1.33, 95% confidence interval (CI): −1.63, −1.03; *Z* = 8.65; *P* < 0.05), reduced NDI scores (MD = −5.54, 95% CI: −9.73, −1.35; *Z* = 2.59; *P* < 0.05), and improved the total effective rate (OR = 0.27, 95% CI: 0.10, 0.70; *Z* = 2.67; *P* < 0.05). Adverse events were not reported in several studies, limiting conclusions on safety. A subgroup analysis revealed that heterogeneity may be related to the variation in combination therapies and treatment course. A sensitivity analysis confirmed the robustness of the results. The overall quality of evidence ranged from very low to moderate.

**Conclusions:**

This study found that thumbtack needle therapy can effectively relieve pain and improve cervical mobility in patients with NP. The reduction in VAS scores reached the level of the minimum clinically important difference, indicating that thumbtack needle treatment for neck pain has a clinically significant impact. In the future, high-quality RCTs are needed to further validate the clinical efficacy of thumbtack needle therapy for NP.

**Systematic Review Registration:**

https://www.crd.york.ac.uk/PROSPERO/view/CRD42025632076, PROSPERO CRD42025632076.

## Introduction

Neck pain (NP) is defined as pain in the cervical spine with or without symptoms in the upper limbs, lasting at least 1 day (1). Its global 1-year rate of incidence ranges from 10.4% to 21.3% (2), making it one of the leading causes of disability (3). Its incidence varies across different regions in China, but the overall prevalence is increasing year by year, with an increasing proportion of cases among younger populations (4). This phenomenon imposes considerable burden on both individuals and socioeconomic systems (5, 6). As stated in the updated clinical practice guidelines formulated by the Orthopedic Section of the American Physical Therapy Association (APTA), currently recommended Western medical treatments for NP include muscle relaxants, non-steroidal anti-inflammatory drugs (NSAIDs), and opioids (7–9). However, some researchers found that these drugs had non-negligible side effects such as dizziness, drowsiness, addiction, constipation, and gastrointestinal and cardiovascular complications (10–12).

In Traditional Chinese Medicine (TCM), conventional therapeutic approaches for NP include various acupuncture treatments, Chinese herbal medicine, massage therapy, and topical Chinese medicinal applications (13). Thumbtack needle, a minimally therapeutic tool in complementary medicine, exerts its effects through transcutaneous stimulation-induced microcurrent generation. This process facilitates the exchange of relevant biochemical substances and activates endogenous opioid peptide release (e.g., endorphins and enkephalins) within the tissue microenvironment, thereby exerting neurohumoral and immunomodulatory effects. These effects on the nervous system can be maintained for clinically determined periods by inserting microfilaments at controlled depths and prolonging retention (14, 15). In addition, this form of therapy is characterized by ease of operation, low discomfort, safety, and prolonged stimulation (16, 17).

Although previous clinical studies showed that thumbtack needle therapy could relieve pain, improve cervical mobility (18–21), and alleviate anxiety and depression symptoms (22), some randomized controlled trials (RCTs) revealed no significant pain-relieving effects of this treatment (23). Notably, the results of these studies were inconsistent, and no systematic evaluation of the therapeutic efficacy and safety profile of thumbtack needle therapy for NP has been conducted. Therefore, this systematic review and meta-analysis aims to quantitatively assess the therapeutic efficacy and safety profile of thumbtack needle therapy in the management of NP, compared with modern pharmacotherapy or physiotherapy methods, using Review Manager 5.3. The findings are expected to provide evidence-based recommendations for clinical decision-making regarding NP interventions.

## Methods

This meta-analysis was performed in accordance with the Preferred Reporting Items for Systematic Review and Meta-Analyses (PRISMA) guidelines (24). The protocol was registered in the International Prospective Register of Systematic Reviews (PROSPERO) under registration number CRD42025632076.

### Eligibility criteria

#### Inclusion criteria

*Patients:* Individuals diagnosed with NP, regardless of age, gender, or nationality, were chosen.

*Interventions:* The intervention group received thumbtack needle therapy. Studies comparing thumbtack needle therapy combined with other therapies against those same therapies alone were also included.

*Comparators:* Control groups received sham thumbtack needle therapies, no treatment, or other therapies.

*Outcomes:* Pain intensity was assessed using the Visual Analog Scale (VAS).

*Study type:* Only RCTs published in Chinese or English were included.

#### Exclusion criteria

Studies were excluded if they met any of the following items: (1) duplicate publications; (2) studies with incomplete or incorrect data; or (3) studies without available full text.

### Search strategies

A systematic literature search was conducted across seven databases from inception to 24 September 2023, including the Cochrane Library, Web of Science, Embase, PubMed, China National Knowledge Infrastructure (CNKI), Wanfang Database, and the Chinese Science and Technology Journal (VIP) Database. Search terms in Embase included the following: (“intradermal needle” OR “thumb-tack needle” OR “press-needle” OR “hypodermic acupuncture” OR “subcutaneous needle” OR “subcutaneous acupuncture”) AND (“pain” OR “ache” OR “burning”). Search strategies for other databases were adapted accordingly and are detailed in Supplementary Table S1.

### Data extraction

Two reviewers (He and Lai) independently screened titles, abstracts, and full texts. Data were extracted using a predefined form, covering authors, publication year, sample size, age, gender, illness duration, interventions, outcome indicators, and adverse events. Disagreements were resolved by discussion with a third reviewer (SC).

### Assessment of risk of bias

Methodological quality was assessed independently by two reviewers (He and Lai) using the Cochrane risk bias assessment tool (25), evaluating the following domains: (1) random sequence generation; (2) allocation concealment; (3) blinding of participants or personnel; (4) blinding of outcome assessment; (5) incomplete outcome data; (6) selective reporting; and (7) other biases (e.g., practitioner experience). Each domain was rated as low, high, or unclear risk. Any discrepancies were resolved through consensus with a third reviewer (SC).

### Quality assessment of evidence

The quality of evidence for each outcome was assessed using the Grading of Recommendations Assessment, Development, and Evaluation (GRADE) system (25) based on five criteria: limitations of the study, inconsistency of results, indirectness, imprecision, and other considerations. Finally, five levels of evidence were identified (26): high, moderate, low, very low, and no evidence. Data extraction was performed independently by two reviewers (JH and PL), with any discrepancies resolved by a third reviewer (SC).

### Statistical analysis

Meta-analysis was performed using Review Manager 5.3. For continuous outcomes, mean difference (MD) with 95% confidence interval (CI) was calculated. For dichotomous outcomes, odds ratio (OR) with 95% CI were conducted. Heterogeneity was evaluated using the I² statistic. A fixed-effects model was applied if *I*^2^ < 50% and *P* > 0.1; a random-effects model was applied if *I*^2^ > 50%, *P* < 0.1.

### Sensitivity, subgroup, and publication bias analysis

Sensitivity analyses were performed utilizing the leave-one-out method. Subgroup analyses were conducted based on different combinations of thumbtack needle with other interventions, types of outcome indicators, and course of thumbtack needle treatment. The funnel plot and Egger's test were used to evaluate publication bias (a value of *P* < 0.05 was considered to indicate significant publication bias among the enrolled studies).

## Results

### Study selection and characteristics

**Figure 1:**
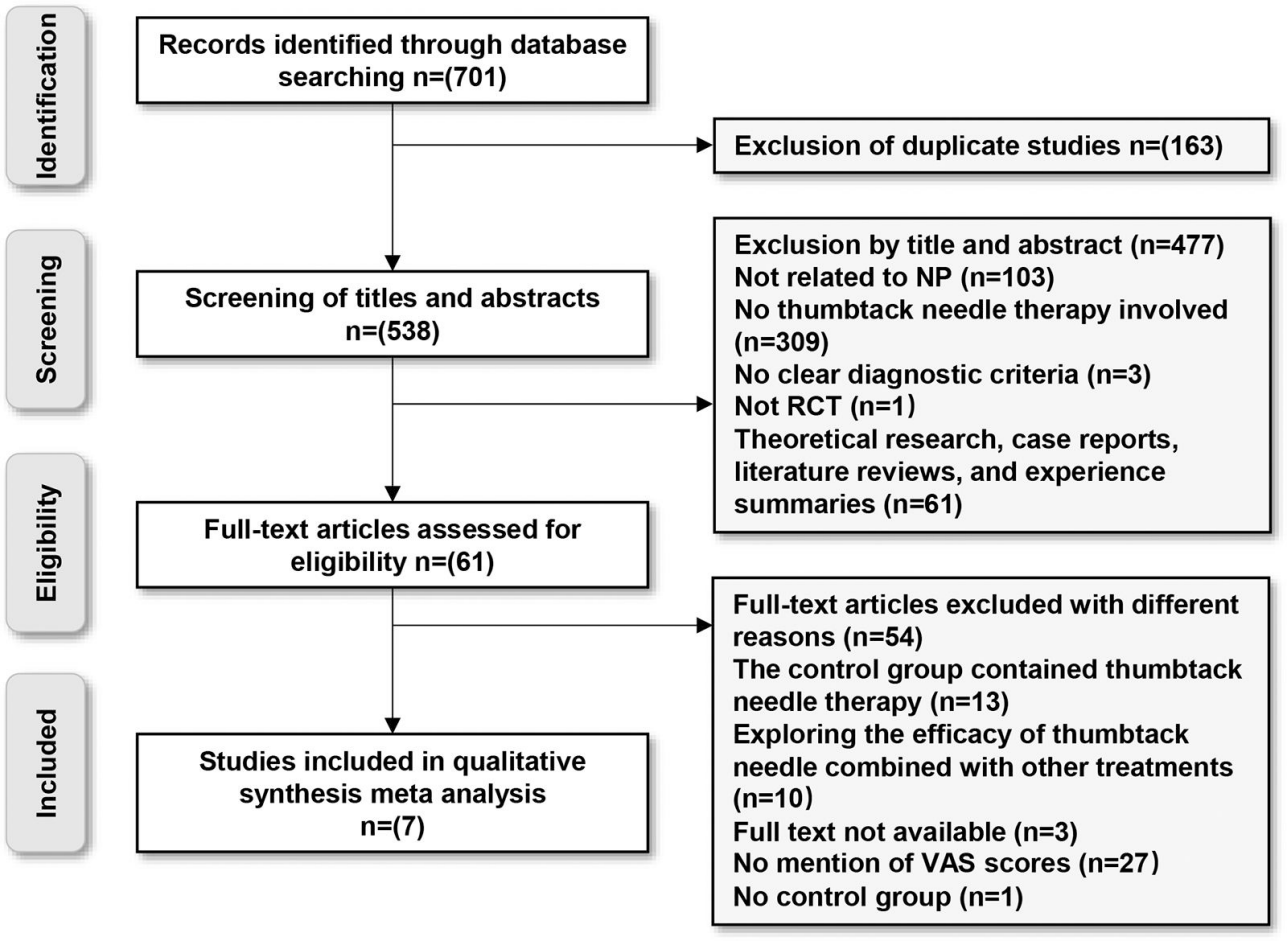
A flow diagram of studies included according to the inclusion and exclusion criteria. NP, neck pain; RCT, randomized controlled trial; VAS, visual analog scale.

The initial search yielded 701 potential studies. After removing duplicates and screening, seven RCTs involving 425 participants were included (Figure 1). Three of these studies (18, 21, 27) did not report illness duration. Detailed characteristics of the included trials are summarized in Table 1.

**Table 1:** Characteristics of the literature.

Study (author/year)	Sample size	Mean age (means ± SD) (years)		Duration (means ± SD) (month)		Interventions		Treatment course	Outcomes
		Experimental	Control	Experimental	Control	Experimental	Control		
Gao (29)	62	36.71 ± 8.63	34.06 ± 7.62	22.68 ± 28.36	16.23 ± 27.99	①	②	2 weeks	VAS, effective rate
Huang (18)	80	33.3 ± 8.8	37.2 ± 11.6	NA	NA	① + ③	③	2 weeks	VAS, NDI
Lou (19)	60	46.47 ± 12.30	45.90 ± 12.85	8.67 ± 5.93	7.67 ± 4.01	① + ④	④	4 weeks	VAS, NDI, effective rate
Su (20)	64	38.79 ± 3.68	38.57 ± 3.44	92.76 ± 15	93.84 ± 15.96	① + ③	③	2 weeks	VAS, NDI
Sun (28)	60	41.10 ± 13.626	44.37 ± 14.187	3.06 ± 3.25	2.69 ± 3.16	① + ⑤	⑤	4 weeks	VAS, effective rate
Wu (21)	59	37.83 ± 10.48	38.53 ± 9.43	NA	NA	① + ⑥	⑥	2.9 weeks	VAS, NDI, effective rate
Yang (27)	40	45.09 ± 2.15	44.94 ± 2.14	NA	NA	① + ⑦	⑦	NA	VAS, effective rate

①, Thumbtack needle; ②, Western medicine; ③, Transcutaneous electrical nerve stimulation (TENS); ④, Chinese medicine; ⑤, Acupuncture; ⑥, Tuina; ⑦, Traditional Chinese medicine (TCM) nursing. NA, not applicable; NDI, neck disability index; SD, standard deviation; VAS, visual analog scale.

### Risk of bias in included studies

Six studies used random tables (18, 20, 21, 27, 28) or computer-generated (29) sequences for randomization and were considered to have low risk in the domain of random sequence generation. One study (19) did not describe the method used, resulting in an unclear risk of bias. None of the included studies described allocation concealment, resulting in an unclear risk across all trials (18–21, 27–29).

**Figure 2:**
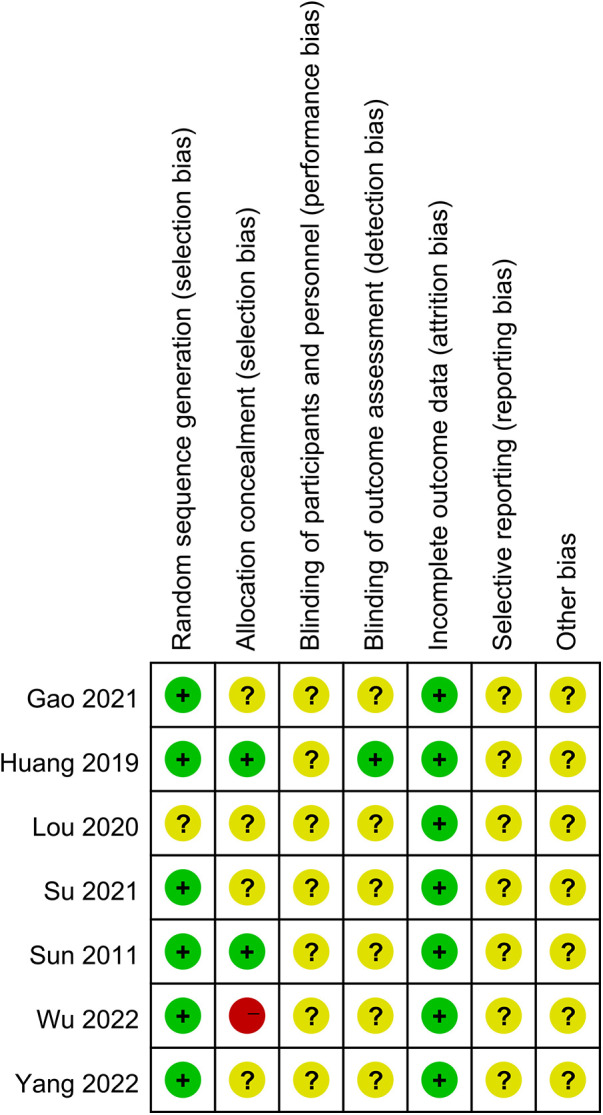
Risk of bias analysis of each included study.

**Figure 3:**
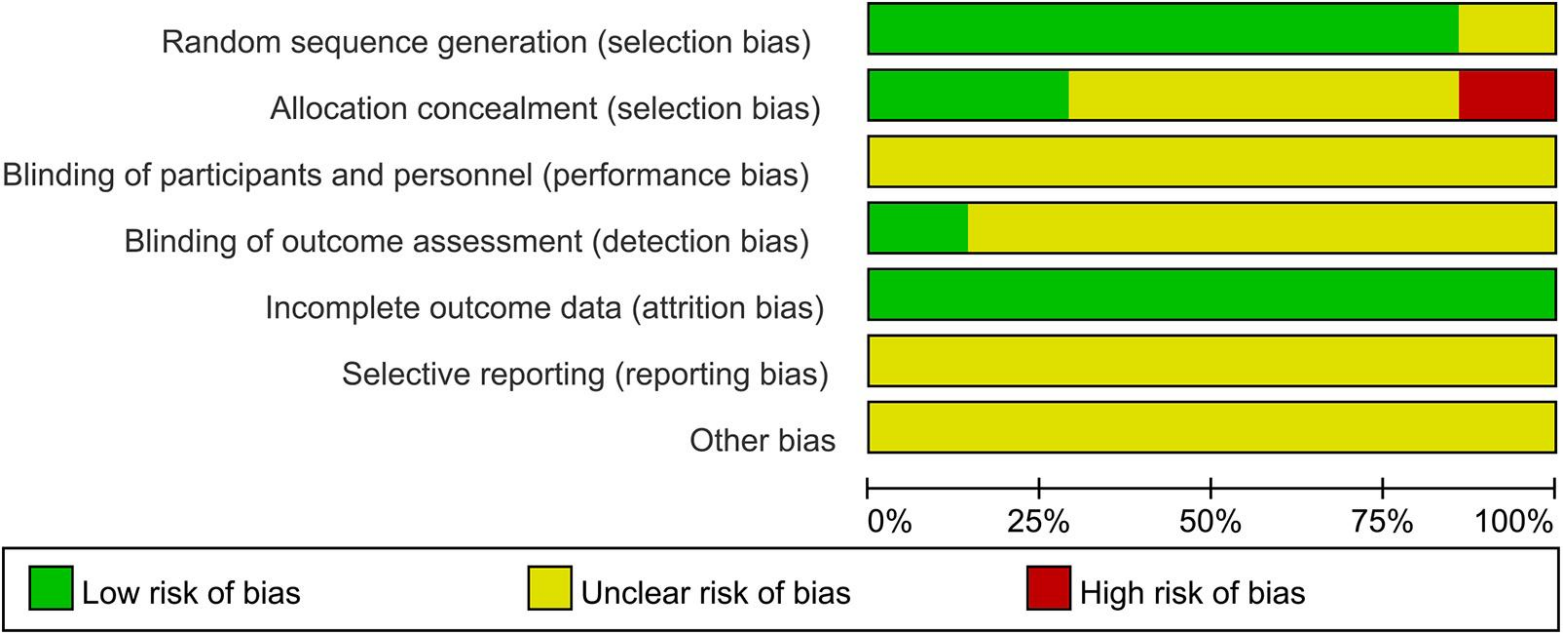
The overall risk of bias analysis of included studies.

Blinding was inconsistently reported. One study (27) explicitly stated that no blinding was implemented, resulting in a high risk of performance and detection bias. The remaining studies (18–21, 28, 29) did not describe their blinding strategies. All studies reported complete outcome data and were rated as low risk for attrition bias. However, selective reporting and other biases were generally unclear across studies (18–21, 27–29), due to insufficient information. Two studies (27, 29) reported dropouts but clarified that these cases did not impact study outcomes. The results of the risk of bias assessment are illustrated in Figures 2, 3.

### Thumbtack needle with other treatments vs. other treatments

#### Visual analog scale scores

**Figure 4:**
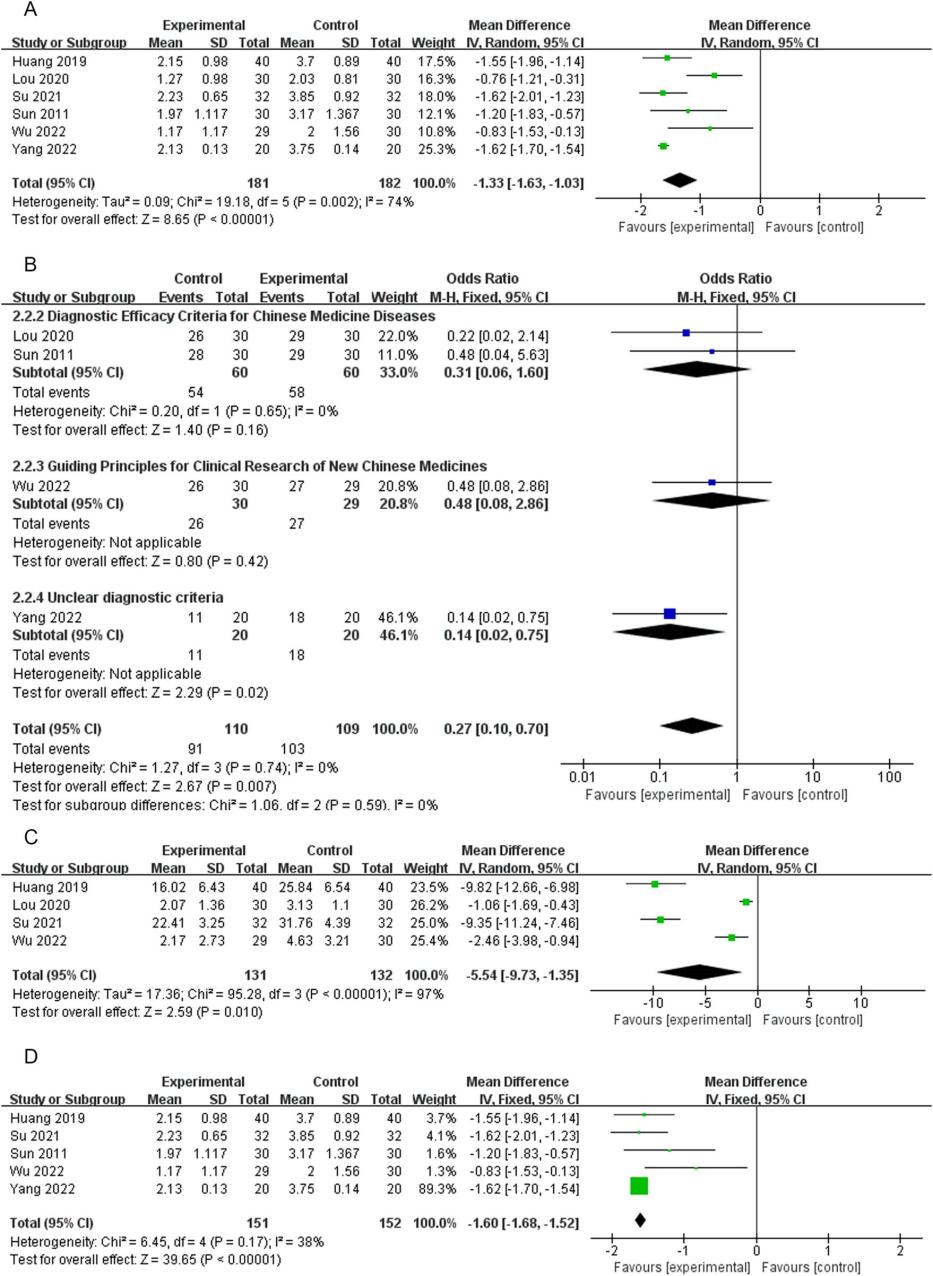
A forest plot of thumbtack needle vs. other treatments on VAS **(A)**, total effective rate **(B)**, NDI **(C)**, and forest plot of thumbtack needle vs. other treatments after removing one study on VAS **(D)** VAS, visual analog scale; NDI, neck disability index.

**Figure 5:**
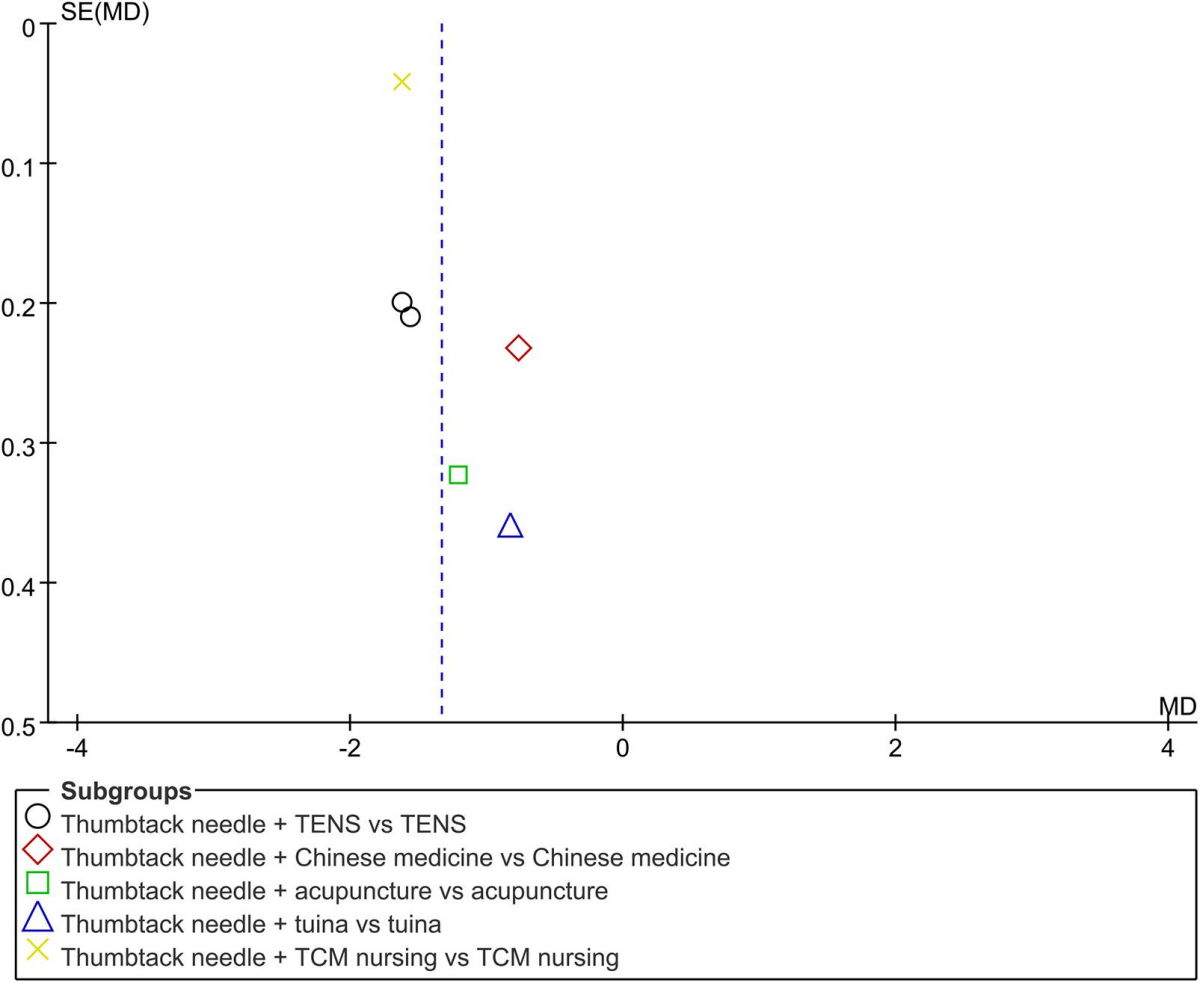
A funnel plot of VAS: scatter indicates the included studies. TCM, traditional Chinese medicine; TENS, transcutaneous electrical nerve stimulation; vs., versus.

**Figure 6:**
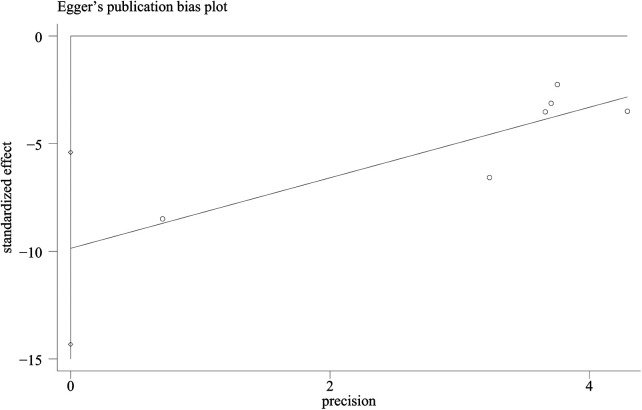
Egger's test plot of VAS: scatter indicates the included studies.

Six RCTs (18–21, 27, 28) reported VAS scores. Low-quality evidence indicated that thumbtack needle therapy significantly reduced VAS scores compared with controls (MD = −1.33, 95% CI: −1.63 to −1.03, *Z* = 8.65, *P* < 0.05), with great heterogeneity among studies (*I*^2^ = 74%) (Figure 4). The sensitivity analyses revealed that if we exclude one study by Lou et al. (19), *I*^2^ decreased from 74% to 38% and MD changed from −1.33 to −1.60. After excluding that study, the combined results of the remaining studies remained stable (Figure 4). The results of funnel plots and Egger's test indicated significant publication bias (*P*_egge*r*_ = 0.04) in the included studies (Figures 5, 6).

#### Total effective rate

Four RCTs (19, 21, 27, 28) reported a significantly total effective rate in the thumbtack needle group with moderate-quality evidence (OR = 0.27, 95% CI: 0.10–0.70, *Z* = 2.67, *P* < 0.05), with no heterogeneity among the studies (*I*^2^ = 0%) (Figure 4).

#### Neck disability index scores

Neck disability index (NDI) scores were reported in four of the RCTs (18–21). There was very low-quality evidence, indicating that thumbtack needle therapy was more effective in improving the cervical range of motion compared with controls (MD = −5.54, 95% CI: −9.73 to −1.35, *Z* = 2.59, *P* < 0.05). However, there was substantial heterogeneity among the studies (*I*^2^ = 97%) (Figure 4).

### Acupuncture vs. active control

The study by Gao (29) compared thumbtack needle with Voltaren (a first-line NSAID). No significant differences were observed in VAS scores (MD = −0.39, 95% CI: −0.95 to 0.17, *Z* = 1.37, *P* > 0.05), total effective rate (OR = 0.31, 95% CI: 0.03–3.17, *Z* = 0.99, *P* > 0.05), and clinical assessment scale for cervical spondylosis (CASCS) (MD = −2.60, 95% CI: −5.60 to 0.40, *Z* = 1.70, *P* > 0.05). However, this study demonstrated a notable enhancement in Northwick Park questionnaire (NPQ) scores (MD = −4.12, 95% CI: −7.91 to −0.33, *Z* = 2.13, *P* < 0.05) (Supplementary Figure S1).

### Subgroup analyses

**Figure 7:**
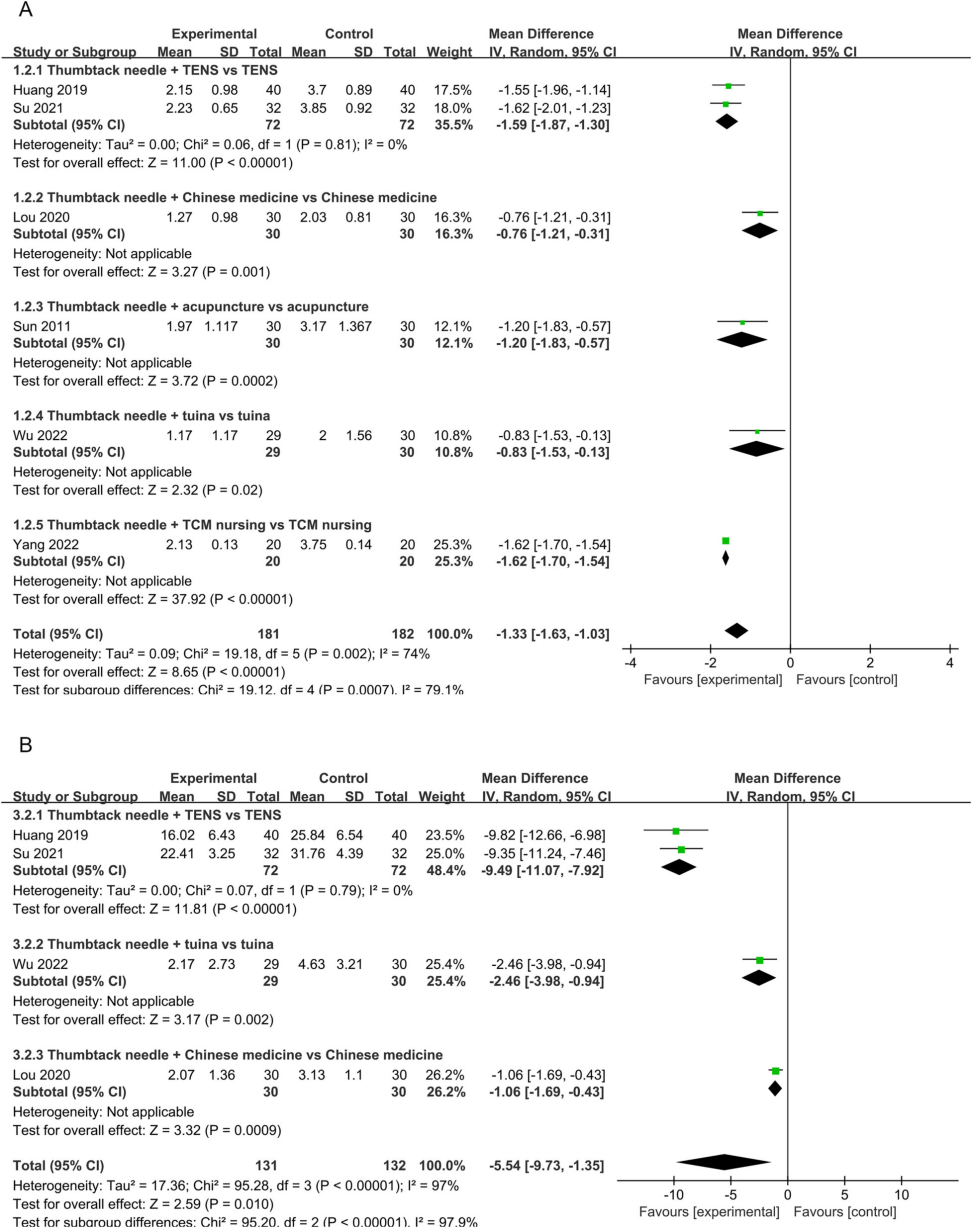
A forest plot of thumbtack needle vs. other treatments on the subgroup of VAS **(A)** and NDI **(B)** TCM, traditional Chinese medicine; TENS, transcutaneous electrical nerve stimulation; VAS, visual analog scale; NDI, neck disability index; vs., versus.

**Figure 8:**
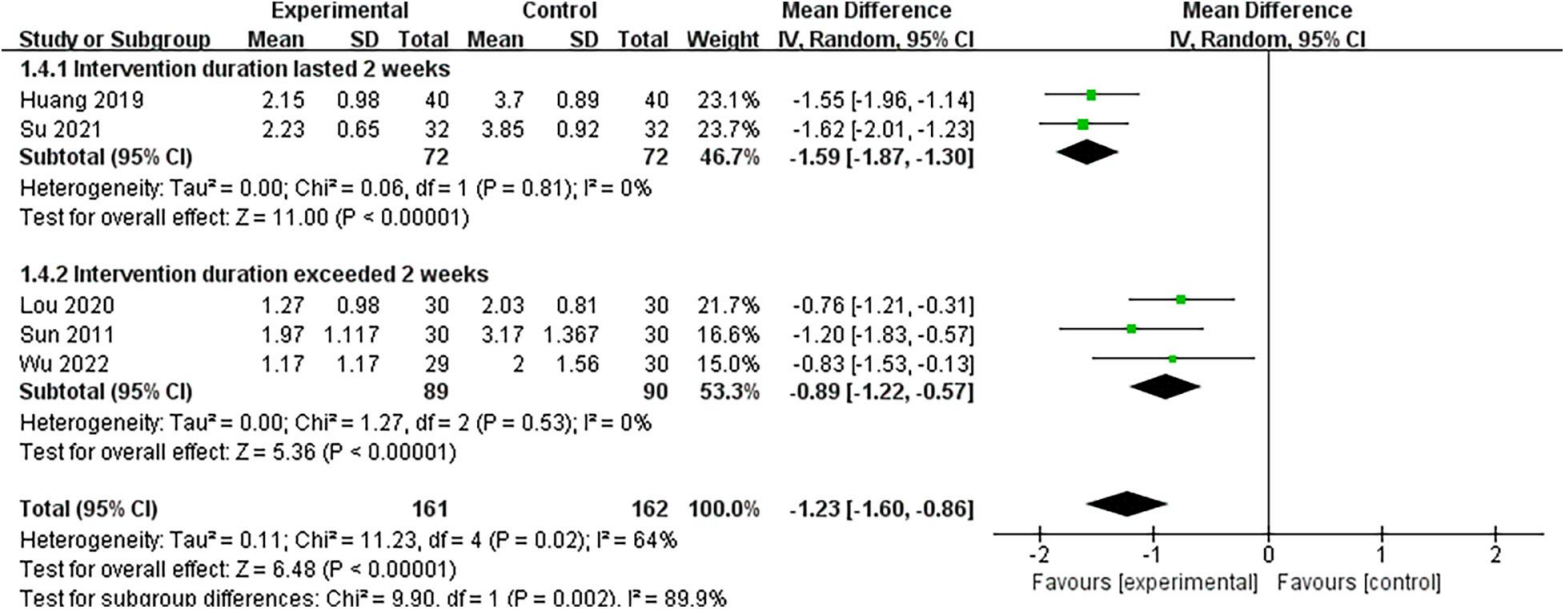
A forest plot of variation in treatment course in the subgroup of VAS.

Subgroup analyses revealed that, compared with TENS alone, the combination of TENS and thumbtack needle therapy significantly reduced VAS scores in two studies (MD = −1.59, 95% CI: −1.87 to −1.30, *P* < 0.05, *I*^2^ = 0%) (18, 20). For example, thumbtack needle therapy plus TENS was more effective than TENS alone (18, 20) (MD = −9.49, 95% CI: −11.07 to −7.92, *P* < 0.05, *I*^2^ = 0%) (Figure 7). For treatment courses lasting 2 weeks or more, the combination of thumbtack needle therapy and other treatments significantly reduced VAS scores compared with other treatments alone in two studies (18, 20) (MD = −1.59, 95% CI: −1.87 to −1.30, *P* < 0.05, *I*^2^ = 0%) and three studies (19, 21, 28) (MD = −0.89, 95% CI: −1.22 to −0.57, *P* < 0.05, *I*^2^ = 0%) (Figure 8).

### Evidence quality

The results indicate an overall low quality of evidence, but with varying certainty ranging from very low to moderate. The quality of evidence for VAS scores was low (downgraded by two levels). The total effective rate fell into the moderate category and the quality of evidence was downgraded by one level. The quality of evidence for the NDI scores was rated as very low (downgraded by three levels). Likewise, the evidence quality of the subgroup analyses was rated down 1–3 levels from low to moderate. Detailed assessments for each group are presented in Table 2.

**Table 2:** Summary of findings and quality of evidence for all outcomes included in this review.

Outcomes	Effect size (95% Cl)	No. of participants (studies)	Quality of the evidence (GRADE)
VAS	MD 1.33 lower (1.63–1.03 lower)	363 (6 RCT)	⊕⊕⊝⊝
			Low
Thumbtack needle + TENS vs. TENS	MD 1.59 lower (1.87 lower–1.3 higher)	144 (2 RCT)	⊕⊕⊕⊝
			Moderate
Thumbtack needle + Chinese medicine vs. Chinese medicine	MD 0.76 lower (1.21–0.31 lower)	60 (1 RCT)	⊕⊕⊝⊝
			Low
Thumbtack needle + acupuncture vs. acupuncture	MD 1.2 lower (1.83–0.57 lower)	60 (1 RCT)	⊕⊕⊕⊝
			Moderate
Thumbtack needle + tuina vs. tuina	MD 0.83 lower (1.53–0.13 lower)	59 (1 RCT)	⊕⊕⊕⊝
			Moderate
Thumbtack needle + TCM nursing vs. TCM nursing	MD 1.62 lower (1.7 lower–1.54 higher)	40 (1 RCT)	⊕⊕⊕⊝
			Moderate
Total clinical effective rate	OR 0.27 (0.1–0.7)	219 (4 RCT)	⊕⊕⊕⊝
			Moderate
Diagnostic efficacy criteria for Chinese	OR 0.31 (0.06–1.60)	120 (2 RCT)	⊕⊕⊕⊝
			Moderate
Guiding principles for clinical research of new Chinese medicines	OR 0.48 (0.08–2.86)	59 (1 RCT)	⊕⊕⊕⊝
			Moderate
Unclear diagnostic criteria	OR 0.14 (0.02–0.75)	40 (1 RCT)	⊕⊕⊕⊝
			Moderate
NDI	MD 5.54 lower (9.73–1.35 lower)	263 (4 RCT)	⊕⊝⊝⊝
			Very Low
Thumbtack needle + TENS vs. TENS	MD 9.49 lower (11.07–7.92 lower)	144 (2 RCT)	⊕⊕⊕⊝
			Moderate
Thumbtack needle + tuina vs. tuina	MD 2.46 lower (3.98–0.94 lower)	59 (1 RCT)	⊕⊕⊕⊝
			Moderate
Thumbtack needle + Chinese medicine vs. Chinese medicine	MD 1.06 lower (1.69–0.43 lower)	60 (1 RCT)	⊕⊕⊝⊝
			Low

CI, confidence interval; GRADE, grading of recommendations assessment, development, and evaluation; MD, mean difference; NDI, neck disability index; OR, odds ratio; RCT, randomized controlled trial; TENS, transcutaneous electrical nerve stimulation; VAS, visual analog scale; vs., versus.

### Safety assessment

Among the seven studies, three (19, 20, 27) did not report adverse events. Four studies (18, 21, 28, 29) explicitly stated that no adverse events occurred.

## Discussion

### Summary of main findings

This meta-analysis provides a comprehensive review of the effectiveness and safety of thumbtack needle therapy for NP. Our findings demonstrate that thumbtack needle therapy significantly reduces pain intensity (VAS scores), improves cervical function (NDI scores), and enhances total treatment effectiveness compared with control interventions. Importantly, these benefits were achieved without any serious adverse events occurring, which suggested that thumbtack needle therapy is a safe complementary intervention.

The therapeutic efficacy of thumbtack needle appears particularly obvious when used in combination with conventional therapies such as TENS. Subgroup analyses confirmed significant additive benefits in both VAS and NDI scores when thumbtack needle was combined with TENS, with no observed heterogeneity (*I*^2^ = 0%), suggesting that the heterogeneity stems from differences in the control group's interventions. Similarly, for a treatment course lasting two weeks or longer, thumbtack needle with other treatments significantly reduced VAS scores compared with other treatments alone, with no observed heterogeneity (*I*^2^ = 0%), indicating that the heterogeneity stems from differences in the course of thumbtack needle treatment. However, overall heterogeneity in the primary analyses, particularly for VAS and NDI, was substantial. Sensitivity analyses identified one study (19) as a major contributor to heterogeneity. Omission of this study substantially reduced the *I*² from 74% to 38% but did not change the overall conclusion, suggesting the stability of the results. This study differed from the others in that it used thumbtack needle therapy with oral medication, while the rest used thumbtack needle combined with physical therapy, likely explaining the inconsistency. Therefore, heterogeneity may primarily arise from a variation in combination therapies or the course of thumbtack needle treatment.

### Quality of evidence and limitations

Although this review included studies examining thumbtack needle therapy in various combinations and the results seemed favorable, the quality of evidence certainty was low. The quality of evidence for the VAS score was rated as low, with a downgrade of two levels due to substantial heterogeneity and high publication bias. The total clinical effectiveness was rated as moderate, with the evidence downgraded by one level due to imprecision in the studies. The NDI score was rated as very low, with the evidence downgraded by three levels due to risk of bias, substantial heterogeneity, and imprecision. The comprehensive standard of evidence ranged from very low to moderate, primarily due to methodological limitations in the included studies, including the following: (1) The diagnostic criteria and control interventions were not standardized across trials. (2) No study included a sham thumbtack needle or blank control group, and only one study (29) compared thumbtack needle therapy with an active pharmacological treatment (Voltaren). This limited our ability to conduct a more rigorous comparative analysis. (3) Potential publication bias was suggested by asymmetric funnel plots and Egger's test, indicating that negative or null-result studies may have been underreported. (4) Blinding was generally inadequate or unreported, and allocation concealment was not described in any of the included trials. These design flaws are known to engender bias in RCTs. (5) Details regarding practitioner qualifications and participants' expectations were also absent, raising concerns about uncontrolled confounders. (6) This study collected data from multiple Chinese and English databases and included all RCTs. However, no English-language studies were included ultimately, introducing substantial regional and publication bias. All of these limitations highlight the need for a cautious interpretation of the results.

To improve the reliability and generalizability of evidence on thumbtack needle therapy for NP, future RCTs should incorporate the following improvements: (1) use of placebo (sham intradermal needle) and blank control groups to isolate specific treatment effects; (2) standardization of diagnostic criteria and intervention protocols; (3) blinding of participants and assessors to minimize bias; (4) transparent reporting of sequence generation and allocation concealment; (5) inclusion of details on practitioner expertise and patient expectations; and (6) larger sample sizes and multicenter designs to improve external validity. Such improvements will contribute to the development of high-quality evidence that can inform clinical guidelines and practice.

## Conclusion

Thumbtack needle therapy appears to be an effective and relatively safe treatment option for patients with NP, with demonstrated advantages in alleviating pain, improving cervical function, and increasing overall treatment efficacy. Importantly, no serious adverse events were reported in the included studies. Clinical studies suggest that the minimal clinically important difference (MCID) for the VAS ranges from 0.9 to 1.44 cm (30–32). This study observed a mean VAS score reduction of −1.33 points, indicating that thumbtack needle therapy achieves statistically and clinically significant pain relief that is perceptible to patients. These findings provide a strong rationale for promoting and applying thumbtack needle therapy in clinical practice. Nevertheless, evidence is currently weakened by methodological flaws and potential publication bias. Therefore, the conclusions drawn from this meta-analysis should be interpreted with caution. To better evaluate the true efficacy and safety of thumbtack needle therapy, future clinical trials should aim to adopt rigorous methodologies such as placebo control, standardized protocols, appropriate blinding, and multicenter randomized controlled designs. These steps will improve the quality and applicability of evidence for integrating thumbtack needle therapy into mainstream clinical practice.

## Data Availability

The original contributions presented in the study are included in the article/Supplementary Material; further inquiries can be directed to the corresponding author.

## References

[1] Hoy D March L Woolf A Blyth F Brooks P Smith E The global burden of neck pain: estimates from the global burden of disease 2010 study. Ann Rheum Dis. (2014) 73(7):1309–15. 10.1136/annrheumdis-2013-204431 24482302

[2] Hoy DG Protani M De R Buchbinder R. The epidemiology of neck pain. Best Pract Res Clin Rheumatol. (2010) 24(6):783–92. 10.1016/j.berh.2011.01.019 21665126

[3] Vos T Allen C Arora M Barber RM Bhutta ZA Brown A Global, regional, and national incidence, prevalence, and years lived with disability for 310 diseases and injuries, 1990–2015: a systematic analysis for the Global Burden of Disease Study 2015. Lancet. (2016) 388(10053):1545–602. 10.1016/S0140-6736(16)31678-6 27733282 PMC5055577

[4] Cui XJ Yao M. Expert consensus on the diagnosis and treatment of cervical spondylosis with integrated traditional Chinese and western medicine. World Chin Med. (2023) 18(07):918–22. 10.3969/j.jssn.1673-7202.2023.07.005

[5] Cohen SP Hooten WM. Advances in the diagnosis and management of neck pain. Br Med J. (2017) 358:j3221. 10.1136/bmj.j3221 28807894

[6] Dieleman JL Cao J Chapin A Chen C Li Z Liu A US Health care spending by payer and health condition, 1996–2016. JAMA. (2020) 323(9):863–84. 10.1001/jama.2020.0734 32125402 PMC7054840

[7] Cohen SP. Epidemiology, diagnosis, and treatment of neck pain. Mayo Clin Proc. (2015) 90(2):284–99. 10.1016/j.mayocp.2014.09.008 25659245

[8] Blanpied PR Gross AR Elliott JM Devaney LL Clewley D Walton DM Neck pain: revision 2017. J Orthop Sports Phys Ther. (2017) 47(7):A1–A83. 10.2519/jospt.2017.0302 28666405

[9] Huang JF Meng Z Zheng XQ Qin Z Sun X-L Zhang K Real-world evidence in prescription medication use among U.S. adults with neck pain. Pain Ther. (2020) 9(2):637–55. 10.1007/s40122-020-00193-1 32940899 PMC7648792

[10] Bindu S Mazumder S Bandyopadhyay U. Non-steroidal anti-inflammatory drugs (NSAIDs) and organ damage: a current perspective. Biochem Pharmacol. (2020) 180:114147. 10.1016/j.bcp.2020.114147 32653589 PMC7347500

[11] See S Ginzburg R. Choosing a skeletal muscle relaxant. Am Fam Physician. (2008) 78(3):365–70. 18711953

[12] Stein C. New concepts in opioid analgesia. Expert Opin Investig Drugs. (2018) 27(10):765–75. 10.1080/13543784.2018.1516204 30148648

[13] Zhang W Li JX Lou BD Ye Y Shi W Li H Clinical practice guidelines in traditional Chinese medicine rehabilitation—cervical spondylosis (Xiangbi). Rehab Med. (2020) 30(5):337–42. 10.3724/SP.J.1329.2020.05002

[14] Qi S Li N. Historical evolution and mechanism of action of Qin-acupuncture. Clin J Chin Med. (2019) 11(11):34–6. 10.3969/j.issn.1674-7860.2019.11.011

[15] Tan MS Xiong FS Wang SH Li Q Yang BC Chen Z. A review of the clinical application of acupuncture in orthopaedic disorders. Xinjiang J Tradit Chin Med. (2025) 43(4):112–5.

[16] Mao LH. Advances in the thumbtack needle clinical research over the last 3 years. Inner Mongolia J Tradit Chin Med. (2017) 36(12):151–2. 10.16040/j.cnki.cn15-1101.2017.12.153

[17] Liu AX Huang Y. Examples of the clinical use of thumbtack needle. Xinjiang J Tradit Chin Med. (2018) 36(3):25–6.

[18] Huang J Zhang C Hu C Luo L. Effect of needle-embedding therapy (press needle) on pain and motor function in patients with nonspecific neck pain: a randomized control trial study. Chin J Rehab Theory Pract. (2019) 25(04):465–71. 10.3969/j.issn.1006-9771.2019.04.018

[19] Lou QH Su XL Li B. Efficiency observation of intradermal needling and modified Gegen decoction on neck type of cervical spondylosis. Shanxi J Tradit Chin Med. (2020) 36(5):33–4.

[20] Su MY Fan DH Liu J Zhang ZN Yuan ZX Lin Y Effect of thumbtack needle assisted treatment of non-specific chronic neck pain on patients' cervical spine function. Pract Clin J Inte Tradit Chin West Med. (2021) 21(13):18–19; +69. 10.13638/j.issn.1671-4040.2021.13.008

[21] Wu YZ. Clinical observation on the treatment of neck type cervical spondylopathy by press needle combined with massage therapy *(master's thesis)*. Fujian University of Traditional Chinese Medicine, Fuzhou, China (2022).

[22] Tang JW Xie F. Progress in the study of the clinical application of press-needle therapy. Tradit Chin Med Res. (2023) 36(1):92–6. 10.3969/j.issn.1001-6910.2023.01.24

[23] Horike K Ukezono M. Efficacy of chronic neck pain self-treatment using press needles: a randomized controlled clinical trial. Front Pain Res (Lausanne). (2024) 5:1301665. 10.3389/fpain.2024.1301665 38586186 PMC10995221

[24] Page MJ McKenzie JE Bossuyt PM Boutron I Hoffmann TC Mulrow CD The PRISMA 2020 statement: an updated guideline for reporting systematic reviews. Br Med J. (2021) 372:n71. 10.1136/bmj.n71 33782057 PMC8005924

[25] Higgins JPT Thomas J Chandler J Cumpston M Li T Page MJ Cochrane Handbook for Systematic Reviews of Interventions version 6.5. Cochrane (2024). Available online at: www.training.cochrane.org/handbook (Accessed August 2024).

[26] Balshem H Helfand M Schünemann HJ Oxman AD Kunz R Brozek J GRADE guidelines: 3. Rating the quality of evidence. J Clin Epidemiol. (2011) 64(4):401–6. 10.1016/j.jclinepi.2010.07.015 21208779

[27] Yang XL. Clinical efficacy on the treatment of cervical spondylotic radiculopathy with the combination of thumbtack needle and Chinese medicine nursing. Chin Sci Technol J Database (Full Text Version) Med Health Care. (2022) 14(1):229–31.

[28] Sun W. Clinical effect observation on intradermal needle with traditional acupuncture treating cervical spondylotic radiculopathy. Guangzhou Univ. of Chinese Medicine (2011).

[29] Gao Y. Clinical observation of the treatment of cervical spondylotic disease with the combination of screw acupuncture and yuanluo point. Beijing Univ. of Chinese Medicine (2021).

[30] Swanson BT Gans MB Cullenberg A Cullenberg EK Cyr R Risigo L. Reliability and diagnostic accuracy of cervicothoracic differentiation testing and regional unloading for identifying improvement after thoracic manipulation in individuals with neck pain. Musculoskelet Sci Pract. (2019) 39:80–90. 10.1016/j.msksp.2018.11.01 30529502

[31] MacDowall A Skeppholm M Robinson Y Olerud C. Validation of the visual analog scale in the cervical spine. J Neurosurg Spine. (2018) 28(3):227–35. 10.3171/2017.5.SPINE1732 29243996

[32] Gallagher EJ Liebman M Bijur PE. Prospective validation of clinically important changes in pain severity measured on a visual analog scale. Ann Emerg Med. 2001;38(6):633–8. 10.1067/mem.2001.118863 11719741

