# Supplementary material for: Efficacy and safety of the thumbtack needle for neck pain: a systematic review and meta-analysis
Source: Front Pain Res (Lausanne). 2026 Jan 12;6:1687334. doi: 10.3389/fpain.2025.1687334 (PMC12832657; doi:10.3389/fpain.2025.1687334)
Supplement: Supplementary file 1 [file Table1.docx]

Supplementary Table S1: Search strategies for databases

| **No.** | **Query results** | **Date** |
| --- | --- | --- |
| Embase |  |  |
| #3 | #1 AND #2 | 24 Sep 2023 |
| #2 | 'thumbtack needle' OR 'press-needle' OR 'hypodermic acupuncture' OR 'subcutaneous needle' OR 'subcutaneous acupuncture' | 24 Sep 2023 |
| #1 | 'pain'/exp OR pain OR ache OR 'burning'/exp OR burning | 24 Sep 2023 |
| Pubmed |  |  |
| #3 | #1 AND #2 | 24 Sep 2023 |
| #2 | (((pain) OR (ache)) OR (burning)) | 24 Sep 2023 |
| #1 | ((((("thumbtack needle") OR ("press-needle")) OR ("hypodermic acupuncture")) OR ("subcutaneous needle")) OR ("subcutaneous acupuncture")) | 24 Sep 2023 |
| Cochrane Library |  |  |
| #3 | #1 AND #2 | 24 Sep 2023 |
| #2 | (pain) OR (ache) OR (burning) | 24 Sep 2023 |
| #1 | (thumbtack needle) OR (press-needle) OR ("hypodermic acupuncture") OR ("subcutaneous needle") OR ("subcutaneous acupuncture") | 24 Sep 2023 |
| Web of Science |  |  |
| #3 | #1 AND #2 | 24 Sep 2023 |
| #2 | (((ALL=(pain)) OR ALL=(ache)) OR ALL=(soreness)) OR ALL=(sore) | 24 Sep 2023 |
| #1 | (((((ALL=("thumbtack needle")) OR ALL=("Intradermal imbedding needle")) OR ALL=("intradermal needles")) OR ALL=("implanting the needle")) OR ALL=("press needle")) OR ALL=("subcutaneous needling") | 24 Sep 2023 |
| Wanfang database |  |  |
|  | 主题:(揿针 OR 皮内针 OR 埋针) AND 主题:(颈椎病 OR 神经根性颈椎病 OR 椎动脉型颈椎病 OR 颈椎间盘突出症 OR 颈神经根综合征 OR 颈椎痛 OR 颈痛) | 24 Sep 2023 |
| CNKI |  |  |
|  | SU %= (颈椎病 + 神经根性颈椎病 + 椎动脉型颈椎病 + 颈椎间盘突出症 + 颈神经（根）综合征 + 颈椎痛 + 颈痛) AND SU %= (揿针 + 皮内针 + 埋针) | 24 Sep 2023 |
| VIP |  |  |
|  | 题名或关键词=颈椎病 OR 颈椎痛 OR 颈痛 OR 颈椎 OR 颈椎间盘 AND 任意字段=揿针 OR 皮内针 OR 埋针 OR 皮下留针 | 24 Sep 2023 |

Abbreviations: CNKI, China national knowledge infrastructure; Sep, September; VIP, Chinese science and technology journal database.
