# Supplementary figures and images for: Efficacy and safety of the thumbtack needle for neck pain: a systematic review and meta-analysis
Source: Front Pain Res (Lausanne). 2026 Jan 12;6:1687334. doi: 10.3389/fpain.2025.1687334 (PMC12832657; doi:10.3389/fpain.2025.1687334)

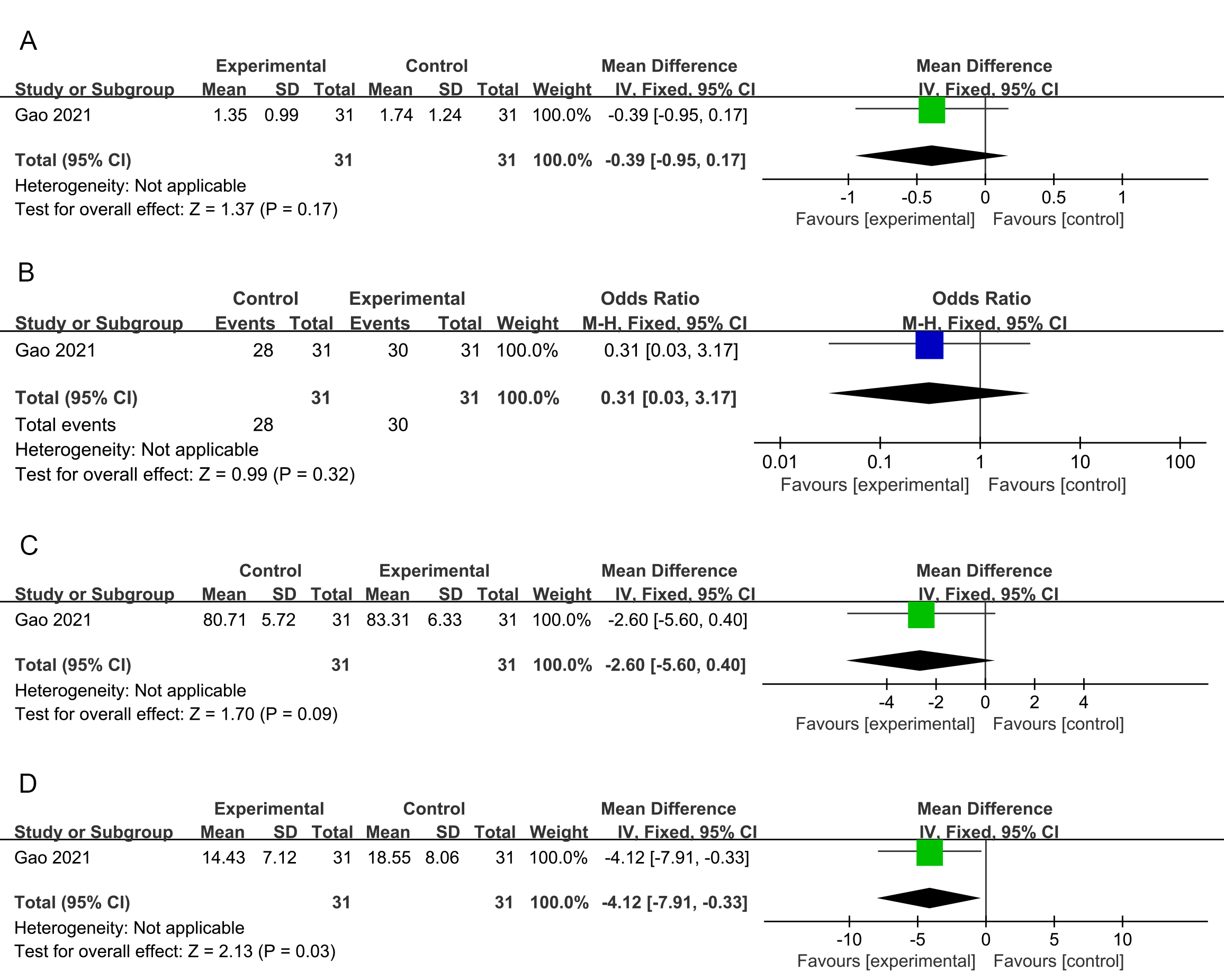

Supplement: Supplementary file 2 [file Image1.tif]
